# 2-(4-Methyl­phen­yl)-2*H*-indazole

**DOI:** 10.1107/S1600536810038948

**Published:** 2010-10-09

**Authors:** Xingqin Zhou, Xiaofen Qin, Jiankang Zhang

**Affiliations:** a Key Laboratory of Nuclear Medicine, Ministry of Health, Jiangsu Key Laboratory of Molecular Nuclear Medicine, Jiangsu Institute of Nuclear Medicine, Wuxi, Jiangsu 214063, People’s Republic of China

## Abstract

The title compound, C_14_H_12_N_2_, was synthesized by the reaction of 4-methyl-*N*-(2-nitro­benz­yl)aniline with tin(II) chloride dihydrate in ethanol at 313 K. The indazole ring system is almost planar with a dihedral angle of 1.58 (10)° between the rings, whereas the plane of the attached *p*-tolyl substituent shows a dihedral angle of 46.26 (5)° with respect to the indazole core.

## Related literature

For the pharmaceutical properties of indazole derivatives, see: Bistochi *et al.* (1981 ▶); Cerecetto *et al.* (2005 ▶); Corsi *et al.* (1976 ▶); Keppler & Hartmann (1994 ▶); Picciola *et al.* (1981 ▶); Rodgers *et al.* (1996 ▶); Sun *et al.* (1997 ▶); Ykeda *et al.* (1979 ▶). For synthetic procedures for indazoles, see: Stadlbauer (2002 ▶).
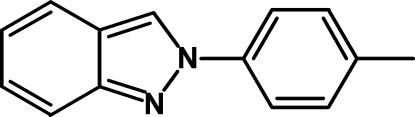


## Experimental

### 

#### Crystal data

C_14_H_12_N_2_
*M* *_r_* = 208.26
Monoclinic, 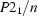

*a* = 12.539 (4) Å
*b* = 6.029 (2) Å
*c* = 14.401 (5) Å
β = 93.636 (5)°
*V* = 1086.4 (6) Å^3^
*Z* = 4
Mo *K*α radiation
μ = 0.08 mm^−1^
*T* = 298 K
0.48 × 0.34 × 0.31 mm

#### Data collection

Bruker SMART CCD area-detector diffractometer
Absorption correction: multi-scan (*SADABS*; Sheldrick, 1996 ▶) *T*_min_ = 0.969, *T*_max_ = 0.980
5372 measured reflections
1915 independent reflections
1236 reflections with *I* > 2σ(*I*)
*R*_int_ = 0.039

#### Refinement

*R*[*F* ^2^ > 2σ(*F* ^2^)] = 0.049
*wR*(*F* ^2^) = 0.140
*S* = 1.03
1911 reflections
145 parameters
H-atom parameters constrained
Δρ_max_ = 0.27 e Å^−3^
Δρ_min_ = −0.26 e Å^−3^

### 

Data collection: *SMART* (Bruker, 1998 ▶); cell refinement: *SAINT* (Bruker, 1999 ▶); data reduction: *SAINT*; program(s) used to solve structure: *SHELXS97* (Sheldrick, 2008 ▶); program(s) used to refine structure: *SHELXL97* (Sheldrick, 2008 ▶); molecular graphics: *SHELXTL* (Sheldrick, 2008 ▶); software used to prepare material for publication: *SHELXTL*.

## Supplementary Material

Crystal structure: contains datablocks global, I. DOI: 10.1107/S1600536810038948/im2233sup1.cif

Structure factors: contains datablocks I. DOI: 10.1107/S1600536810038948/im2233Isup2.hkl

Additional supplementary materials:  crystallographic information; 3D view; checkCIF report

## Figures and Tables

**Fig. 1.**
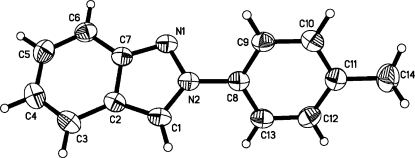
The molecular structure of (I), showing 40% probability displacement ellipsoids and the atom-numbering scheme.

**Fig. 2.**
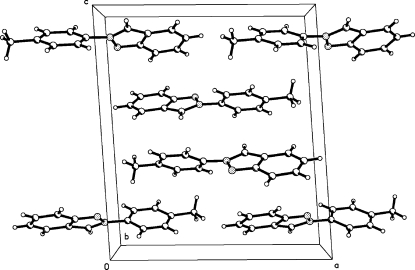
The crystal packing of (I).

## supplementary crystallographic information

### Comment

Indazole is well known as an aza analogue of indole, and a number of indazole derivatives have powerful pharmacological activities including anti-inflammatory (Bistochi *et al.*, 1981; Picciola *et al.*, 1981), antitumor (Keppler & Hartmann, 1994), anti-HIV (Sun *et al.*, 1997; Rodgers *et al.*, 1996), antidepressant (Ykeda *et al.*, 1979), contraceptive activities (Corsi *et al.*, 1976) as well as anti-aggregatory, and vasorelaxant activity by NO release (Cerecetto *et al.*, 2005). Different approaches to the synthesis of 2-substituted indazoles have been reported (Stadlbauer, 2002). However, many of these still suffer from drawbacks as unsatisfactory yields, long reaction time and high temperature. Therefore, the development of more efficient methods for preparation of this kind of compounds is still an active research area.

We report here the crystal structure of the title compound, (I), which was synthesized by the reaction of 4-methyl-*N*-(2-nitrobenzyl)aniline with tin (II) chloride dihydrate using ethanol as solvent at 313 K.

In (I), the pyrazole ring (C1/C2/C7/N1/N2) is a new formed ring. The dihedral angle between the C1/C2/C7/N1/N2 plane and the C2/C3/C4/C5/C6/C7 plane is 1.58 (10)°, so the indazole ring shows an almost perfectly planar conformation. The dihedral angle between the C2/C3/C4/C5/C6/C7 plane and the C8/C9/C10/C11/C12/C13 of the *p*-tolyl substituent plane is 46.26 (5)°.

### Experimental

The title compound, (I), was prepared by the reaction of 4-methyl-*N*-(2-nitrobenzyl)aniline (3 mmol) and tin (II) chloride dihydrate (6 mmol) in ethanol (20 ml) at 313 K (yield: 40%). Crystals of (I) suitable for X-ray diffraction were obtained by slow evaporation of an ethanolic solution. ^1^H NMR (DMSO-d_6_, δ): 2.39 (3*H*, s, CH_3_), 7.09–7.12 (1*H*, m, ArH), 7.29–7.33 (1*H*, m, ArH), 7.40 (2*H*, d, J = 8.4 Hz, ArH), 7.71 (1*H*, d, J = 8.8 Hz, ArH), 7.77 (1*H*, d, J = 8.8 Hz, ArH), 7.98 (2*H*, d, J = 8.4 Hz, ArH), 9.06 (1*H*, s, CH). ^13^C NMR (DMSO-d_6_, δ): 20.69, 117.56, 120.27, 121.01, 121.45, 122.13, 122.58, 126.77, 130.23, 137.53, 137.91, 148.98.

### Refinement

The C-bound H atoms were placed in calculated positions, with C—H = 0.93 or 0.96 Å, and included in the final cycles of refinement using a riding model, with *U*_iso_(H) = 1.2–1.5(methyl) *U*_eq_(C).

### Crystal data

**Table:**

C_14_H_12_N_2_	*F*(000) = 440
*M**_r_* = 208.26	*D*_x_ = 1.273 Mg m^−^^3^
Monoclinic, *P*2_1_/*n*	Mo *K*α radiation, λ = 0.71073 Å
Hall symbol: -P 2yn	Cell parameters from 1425 reflections
*a* = 12.539 (4) Å	θ = 2.8–24.4°
*b* = 6.029 (2) Å	µ = 0.08 mm^−^^1^
*c* = 14.401 (5) Å	*T* = 298 K
β = 93.636 (5)°	Prism, colorless
*V* = 1086.4 (6) Å^3^	0.48 × 0.34 × 0.31 mm
*Z* = 4	

### Data collection

**Table:**

Bruker SMART CCD area-detector diffractometer	1915 independent reflections
Radiation source: fine-focus sealed tube	1236 reflections with *I* > 2σ(*I*)
graphite	*R*_int_ = 0.039
phi and ω scans	θ_max_ = 25.0°, θ_min_ = 2.2°
Absorption correction: multi-scan (*SADABS*; Sheldrick, 1996)	*h* = −12→14
*T*_min_ = 0.969, *T*_max_ = 0.980	*k* = −7→7
5372 measured reflections	*l* = −17→15

### Refinement

**Table:**

Refinement on *F*^2^	Primary atom site location: structure-invariant direct methods
Least-squares matrix: full	Secondary atom site location: difference Fourier map
*R*[*F*^2^ > 2σ(*F*^2^)] = 0.049	Hydrogen site location: inferred from neighbouring sites
*wR*(*F*^2^) = 0.140	H-atom parameters constrained
*S* = 1.03	*w* = 1/[σ^2^(*F*_o_^2^) + (0.0692*P*)^2^ + 0.1862*P*] where *P* = (*F*_o_^2^ + 2*F*_c_^2^)/3
1911 reflections	(Δ/σ)_max_ < 0.001
145 parameters	Δρ_max_ = 0.27 e Å^−^^3^
0 restraints	Δρ_min_ = −0.26 e Å^−^^3^

### Special details

**Table:**

Geometry. All e.s.d.'s (except the e.s.d. in the dihedral angle between two l.s. planes) are estimated using the full covariance matrix. The cell e.s.d.'s are taken into account individually in the estimation of e.s.d.'s in distances, angles and torsion angles; correlations between e.s.d.'s in cell parameters are only used when they are defined by crystal symmetry. An approximate (isotropic) treatment of cell e.s.d.'s is used for estimating e.s.d.'s involving l.s. planes.
Refinement. Refinement of *F*^2^ against ALL reflections. The weighted *R*-factor *wR* and goodness of fit *S* are based on *F*^2^, conventional *R*-factors *R* are based on *F*, with *F* set to zero for negative *F*^2^. The threshold expression of *F*^2^ > σ(*F*^2^) is used only for calculating *R*-factors(gt) *etc*. and is not relevant to the choice of reflections for refinement. *R*-factors based on *F*^2^ are statistically about twice as large as those based on *F*, and *R*- factors based on ALL data will be even larger.

### Fractional atomic coordinates and isotropic or equivalent isotropic displacement parameters (Å^2^)

**Table:**

	*x*	*y*	*z*	*U*_iso_*/*U*_eq_	
N1	0.57580 (13)	0.0040 (3)	0.35143 (12)	0.0442 (5)	
N2	0.55544 (13)	0.2100 (3)	0.38504 (11)	0.0420 (5)	
C1	0.64349 (16)	0.3229 (4)	0.41472 (14)	0.0457 (6)	
H1	0.6458	0.4648	0.4401	0.055*	
C2	0.72979 (16)	0.1879 (4)	0.40029 (13)	0.0433 (5)	
C3	0.84218 (17)	0.2057 (4)	0.41335 (16)	0.0566 (7)	
H3	0.8738	0.3327	0.4393	0.068*	
C4	0.90270 (19)	0.0330 (5)	0.38714 (17)	0.0631 (7)	
H4	0.9767	0.0421	0.3958	0.076*	
C5	0.85600 (18)	−0.1603 (4)	0.34703 (16)	0.0573 (7)	
H5	0.9002	−0.2748	0.3295	0.069*	
C6	0.74854 (17)	−0.1839 (4)	0.33324 (15)	0.0490 (6)	
H6	0.7188	−0.3122	0.3067	0.059*	
C7	0.68372 (16)	−0.0084 (3)	0.36036 (13)	0.0409 (5)	
C8	0.44707 (16)	0.2872 (3)	0.38157 (13)	0.0417 (5)	
C9	0.36778 (16)	0.1513 (4)	0.41005 (14)	0.0473 (6)	
H9	0.3846	0.0112	0.4338	0.057*	
C10	0.26299 (17)	0.2226 (4)	0.40340 (15)	0.0516 (6)	
H10	0.2097	0.1297	0.4231	0.062*	
C11	0.23572 (17)	0.4304 (4)	0.36780 (15)	0.0486 (6)	
C12	0.31752 (18)	0.5648 (4)	0.34099 (16)	0.0538 (6)	
H12	0.3012	0.7054	0.3177	0.065*	
C13	0.42283 (18)	0.4963 (4)	0.34777 (15)	0.0508 (6)	
H13	0.4767	0.5901	0.3298	0.061*	
C14	0.12148 (18)	0.5069 (5)	0.35741 (19)	0.0705 (8)	
H14A	0.0757	0.3925	0.3788	0.106*	
H14B	0.1025	0.5382	0.2931	0.106*	
H14C	0.1133	0.6386	0.3937	0.106*	

### Atomic displacement parameters (Å^2^)

**Table:**

	*U*^11^	*U*^22^	*U*^33^	*U*^12^	*U*^13^	*U*^23^
N1	0.0509 (12)	0.0386 (11)	0.0432 (10)	−0.0059 (8)	0.0047 (8)	−0.0025 (8)
N2	0.0471 (10)	0.0377 (10)	0.0415 (10)	−0.0048 (8)	0.0052 (8)	−0.0015 (8)
C1	0.0542 (13)	0.0396 (12)	0.0435 (12)	−0.0105 (11)	0.0031 (10)	−0.0045 (10)
C2	0.0475 (13)	0.0466 (13)	0.0358 (11)	−0.0073 (10)	0.0036 (9)	0.0012 (10)
C3	0.0516 (14)	0.0626 (16)	0.0550 (15)	−0.0127 (12)	−0.0006 (11)	−0.0055 (12)
C4	0.0455 (14)	0.0786 (19)	0.0649 (16)	−0.0015 (13)	0.0008 (11)	0.0010 (14)
C5	0.0574 (16)	0.0582 (16)	0.0569 (15)	0.0101 (12)	0.0085 (11)	0.0021 (12)
C6	0.0571 (14)	0.0443 (13)	0.0462 (13)	−0.0015 (11)	0.0089 (10)	0.0016 (10)
C7	0.0474 (13)	0.0419 (13)	0.0338 (11)	−0.0041 (10)	0.0061 (9)	0.0037 (9)
C8	0.0481 (12)	0.0409 (13)	0.0360 (11)	−0.0024 (10)	0.0034 (9)	−0.0015 (9)
C9	0.0538 (14)	0.0410 (13)	0.0471 (13)	−0.0047 (11)	0.0024 (10)	0.0072 (10)
C10	0.0494 (14)	0.0526 (15)	0.0530 (14)	−0.0080 (11)	0.0044 (10)	0.0026 (11)
C11	0.0513 (13)	0.0533 (15)	0.0408 (12)	0.0021 (11)	−0.0006 (10)	−0.0063 (11)
C12	0.0639 (16)	0.0453 (14)	0.0516 (14)	0.0050 (12)	−0.0002 (11)	0.0034 (11)
C13	0.0569 (15)	0.0437 (14)	0.0523 (14)	−0.0078 (11)	0.0064 (10)	0.0051 (11)
C14	0.0573 (16)	0.082 (2)	0.0707 (17)	0.0118 (14)	−0.0054 (12)	−0.0070 (15)

### Geometric parameters (Å, °)

**Table:**

N1—C7	1.353 (2)	C6—H6	0.9300
N1—N2	1.362 (2)	C8—C9	1.371 (3)
N2—C1	1.344 (2)	C8—C13	1.378 (3)
N2—C8	1.434 (3)	C9—C10	1.380 (3)
C1—C2	1.380 (3)	C9—H9	0.9300
C1—H1	0.9300	C10—C11	1.388 (3)
C2—C3	1.414 (3)	C10—H10	0.9300
C2—C7	1.422 (3)	C11—C12	1.381 (3)
C3—C4	1.356 (3)	C11—C14	1.503 (3)
C3—H3	0.9300	C12—C13	1.381 (3)
C4—C5	1.411 (3)	C12—H12	0.9300
C4—H4	0.9300	C13—H13	0.9300
C5—C6	1.357 (3)	C14—H14A	0.9600
C5—H5	0.9300	C14—H14B	0.9600
C6—C7	1.405 (3)	C14—H14C	0.9600
			
C7—N1—N2	103.03 (16)	C9—C8—C13	120.3 (2)
C1—N2—N1	113.95 (17)	C9—C8—N2	119.91 (19)
C1—N2—C8	127.16 (19)	C13—C8—N2	119.78 (19)
N1—N2—C8	118.82 (16)	C8—C9—C10	119.9 (2)
N2—C1—C2	106.85 (19)	C8—C9—H9	120.1
N2—C1—H1	126.6	C10—C9—H9	120.1
C2—C1—H1	126.6	C9—C10—C11	121.2 (2)
C1—C2—C3	136.0 (2)	C9—C10—H10	119.4
C1—C2—C7	104.43 (17)	C11—C10—H10	119.4
C3—C2—C7	119.5 (2)	C12—C11—C10	117.6 (2)
C4—C3—C2	118.4 (2)	C12—C11—C14	120.8 (2)
C4—C3—H3	120.8	C10—C11—C14	121.6 (2)
C2—C3—H3	120.8	C11—C12—C13	121.9 (2)
C3—C4—C5	121.5 (2)	C11—C12—H12	119.0
C3—C4—H4	119.2	C13—C12—H12	119.0
C5—C4—H4	119.2	C8—C13—C12	119.1 (2)
C6—C5—C4	121.9 (2)	C8—C13—H13	120.4
C6—C5—H5	119.0	C12—C13—H13	120.4
C4—C5—H5	119.0	C11—C14—H14A	109.5
C5—C6—C7	117.8 (2)	C11—C14—H14B	109.5
C5—C6—H6	121.1	H14A—C14—H14B	109.5
C7—C6—H6	121.1	C11—C14—H14C	109.5
N1—C7—C6	127.45 (19)	H14A—C14—H14C	109.5
N1—C7—C2	111.74 (18)	H14B—C14—H14C	109.5
C6—C7—C2	120.80 (19)		
